# Effective volume of rebreathed air during breathing with facepieces increases with protection class and decreases with ambient airflow

**DOI:** 10.1371/journal.pone.0299919

**Published:** 2024-03-21

**Authors:** Helen Ngo, Johannes Spaeth, Stefan Schumann

**Affiliations:** Faculty of Medicine, Department of Anesthesiology and Critical Care, Medical Center-University of Freiburg, University of Freiburg, Freiburg, Germany; VIT University, INDIA

## Abstract

Wearing facepieces is discussed in the context of increasing the volume of rebreathed air. We hypothesized that rebreathed air volume increases with increasing filtering facepiece (FFP) class and that persons breathing via facepieces compensate for the additional dead-space. We have determined the effective amount of rebreathed air for a surgical masks and FFP2 and FFP3 respirators in a physical model and determined tidal volumes, breathing frequency, blood oxygen saturation, and transcutaneously measured blood carbon dioxide partial pressure (PCO_2_) in lung-healthy subjects breathing without and with facepieces at rest and during exercising on a recumbent ergometer. Rebreathed air volume increased with the facepieces’ protection class and with increasing inspiration volume by 45 ± 2 ml to 247 ± 1 ml. Ambient airflow reduced rebreathed air volume by 17% up to 100% (all p < 0.001). When wearing facepieces, subjects increased tidal volume (p < 0.001) but not breathing frequency. Oxygen saturation was not influenced by facepieces. With FFP3 respirators PCO_2_ increased by up to 3.2 mmHg (p < 0.001) at rest but only up to 1.4 mmHg (p < 0.001) when exercising. Discomfort of breathing increased with increasing protection class of the facepiece but was consistently perceived as tolerable. We conclude that the amount of rebreathed air increases with increasing protection class of facepieces. Healthy adults were capable to compensate the facepieces’ dead-space by adapting tidal volume at rest and during physical activity; thereby they tolerated moderate increases in PCO_2_. Ambient airflow may considerably reduce the amount of facepiece related rebreathed air.

## Introduction

Wearing a facepiece is common practice in various occupational settings, particularly in medical and laboratory working environment. In response to the COVID-19 pandemic, facepieces, i.e. surgical masks and respirators, have become a major means for protecting others but also oneself from respiratory infections in public and in occupational settings. However, wearing facepieces bears potential implications on the individual subject. Among the most discussed is rebreathing of carbon dioxide (CO_2_) [1,2]. The filter material of the facepiece establishes an additional space between the anatomical airways and the ambient, restricting gas exchange. From the physics described by Fick’s law, it follows that the density of the filter material defines the extent of substance exchange across. Transferring these principles into the context of wearing facepieces, it can be assumed that different types of facepieces generate varying degrees of additional functional dead space, influencing the potential for CO_2_ rebreathing. Moreover, clearance from CO_2_ at the ambient side, e.g. by ambient airflow, may have an impact on the effective amount of rebreathed CO_2_.

Interestingly, the still scarce evidence concerning physiological effects of humans wearing facepieces suggests that levels of CO_2_ remain within relatively normal ranges at rest and during physical activity in both adults [1] and in young children [3]. On the other hand, patients with COPD in an advanced stage of their disease could not cope with the additional resistive load [4]. Whereas it is relatively straightforward to monitor respiration frequency to understand how individuals compensate for the required additional breathing volume, measuring tidal volume poses a challenge since standard volume measurements cannot be applied with a facepiece in situ.

We therefore aimed to investigate the amount of effectively rebreathed air caused by surgical masks and respirators, and the effects of wearing these on oxygenation and CO_2_ elimination in healthy subjects. We hypothesised that surgical masks and respirators increase the amount of rebreathed air, and that lung-healthy adults breathing via facepieces compensate for the additional dead-space by increasing tidal volume. We further hypothesised that ambient airflow reduces the amount of rebreathed air. Therefore, we determined the effective amount of rebreathed air for a surgical mask and for respirators of filtering facepiece (FFP) classes FFP2 and FFP3 in a physical model with and without ambient airflow, and we determined tidal volumes, oxygen saturation (SaO_2_) and transcutaneously measured blood CO_2_ partial pressure (PCO_2_) in adult lung-healthy subjects breathing without and with facepieces at rest and during physical activity.

## Materials and methods

### Bench study

**Fig 1:**
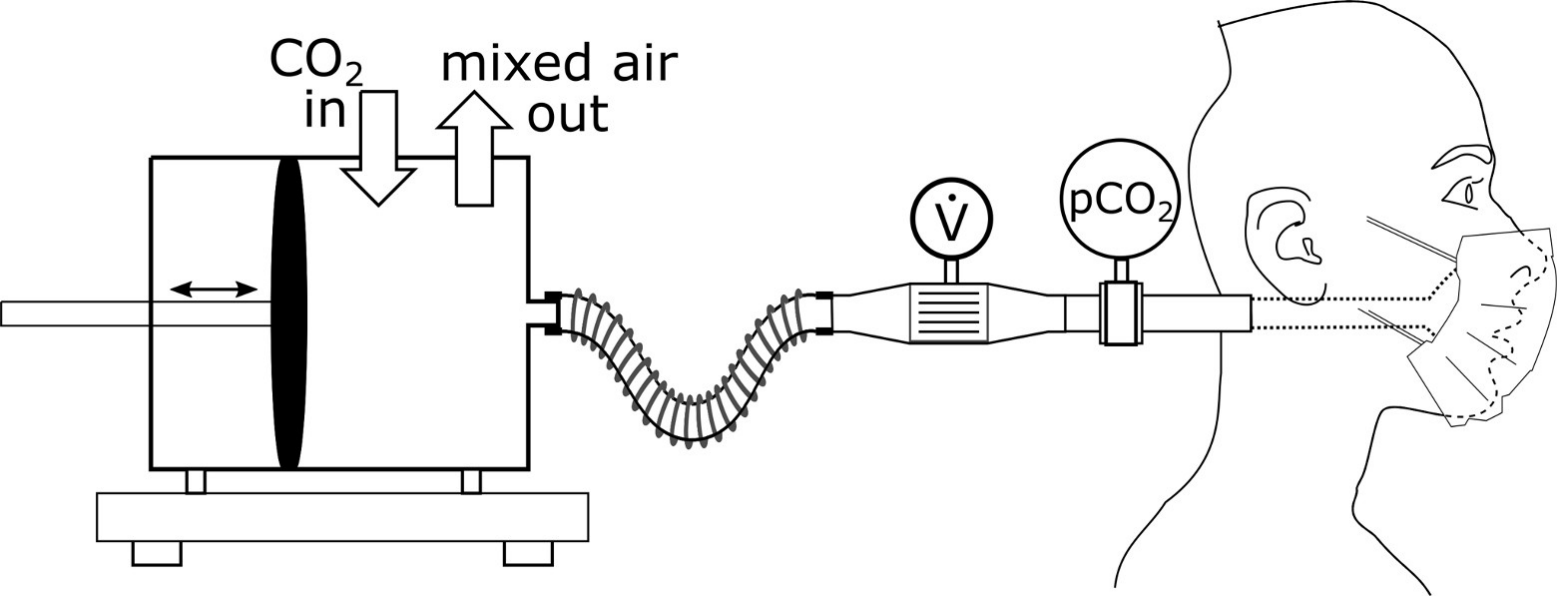
Schematic of the setup for determining effective volume of rebreathed air. A computer controlled piston pump generated a sinusoidal breathing flow with CO_2_ containing air. A continuous CO_2_ inflow and mixed air outflow resulted in an equilibrated CO_2_ partial pressure in the expired air while maintaining volume consistency. The flow rate ($\dot{V}$) was measured via a pneumotachograph and the air’s CO_2_ partial pressure (pCO_2_) via an infrared mainstream sensor.

For determination of the facepiece-dependent amount of rebreathed air we simulated inspiration of ambient air and expiration of CO_2_ containing air in a physical model of airways and facial contour (Fig 1). The bench experiments were performed at a site of a laboratory room of 32 m^2^, shielded against airflow from the air condition which provided a room temperature of 21°C and humidity of 40%. The simulation of breathing was achieved via a self-developed piston pump which was driven by a computer controlled linear motor. With the piston pump, gas was shifted along a standard hose towards a face mannequin with modelled nasal and oral gas orifices. The face mannequin was a 3D printed contour of a computer-scanned face [5]. For measuring PCO_2_, a mainstream sensor (CO_2_ Sensor 6870300, Drägerwerk, Lübeck, Germany) and for measuring flow rate, a pneumotachograph (Type 2, Dr. Fenyves & Gut, Hechingen, Germany) were placed within the gas-carrying hose. For the measurements the facepieces were placed on the face mannequin using the straps as intended (i.e. behind the ears or around the head). The facepiece was placed on the face mannequin as intended and the dedicated nose wire was gently pressed along the contour of the bridge of the nose for proper fit. The PCO_2_ signal and the signal from the flow sensor were digitalised at a sampling rate of 250 1/s. Expiration gas was mixed by continuous insufflation of CO_2_ into the piston pump’s lumen and drawing the same amount of mixed gas to achieve volume consistency. Thereby, the CO_2_ inflow was set with the intention to achieve end tidal CO_2_ partial pressure (PetCO_2_) in the physiological range. The volumes of rebreathed air were determined for the dead-space of the setup (referring to the CO_2_ sensor’s position within the gas-carrying hose) without facepiece, with a surgical mask, with 4 respirators according to FFP2 and with two respirators according to FFP3 protection class (information on types are presented in Table 1, images of the facepieces are provided as supporting information). Breathing was simulated with low and high tidal volumes (450 ml and 950 ml) at a frequency of 12, respectively 8 breaths per minute with the intention to mimic normal and deep breathing at rest. In order to determine the effects of air circulation, e.g. during walking or in presence of ambient airflow caused by air-conditioning, the experiments were performed without and with a constant ambient frontal airflow towards the facepiece. The airflow of 0.2 m/s was generated by a fan (Turbo Fan, Honeywell, Charlotte, NC) set to level 2 and placed at a distance of 1 m to the facepiece. Each experiment was repeated three times in each condition after complete dismantling and reassembly of the setup.

**Table 1:** Manufacturer information on investigated surgical mask and respirators.

Type	Manufacturer	Protection class	Special features
1A3100 Earloop 3-PLY	Dochem Industries Co., Ltd. Shanghai, China	Surgical mask	Flat-fold, multiple horizontal folds; Non-adjustable ear loops
Aura 1862+	3M, Deutschland GmbH, Neuss, Germany	FFP2	Flat-fold, two horizontal folds; Non-adjustable head strap
Aura 1872V+	3M	FFP2	Flat-fold, two horizontal folds; Expiration valve; Non-adjustable head strap
TY0929V	Te Yin, Suzhou, China	FFP2	Cup-shaped; Expiration valve; Adjustable head strap
atemious pro Art. 2001	Univent medical GmbH, Villingen-Schwenningen, Germany	FFP2	flat-fold, single vertical fold; Non-adjustable ear loops
Aura 1863+	3M	FFP3	flat-fold, two horizontal folds; Non-adjustable head strap
silv-Air 7312	uvex, Wels, Austria	FFP3	Cup-shaped; Expiration valve; Adjustable head strap

In a subsequent offline analysis, the volume of rebreathed air (V_rebr_) was calculated using self-written scripts (Matlab, R2020a, Natick, MA) based on Bohr’s approach [6] which was modified to determine V_rebr_ adapted to our setup, i.e. from measurement of PCO_2_ within the simulated person’s airways during the inspiration:

$$V_{rebr}=V_{T}\times \frac{\bar{PinspCO_{2}}}{PetCO_{2}},$$ (1)

with *V*_*T*_ representing tidal volume and $\bar{PinspCO_{2}}$ representing the mean of the PCO_2_-volume curve during the inspiration. To determine the amount of rebreathed air caused by the facepieces, the amount of rebreathed air of the setup without facepiece (determined as 156 ml) was subtracted from the totally determined amount of rebreathed air with the respective facepiece.

### Study in healthy subjects

**Fig 2:**
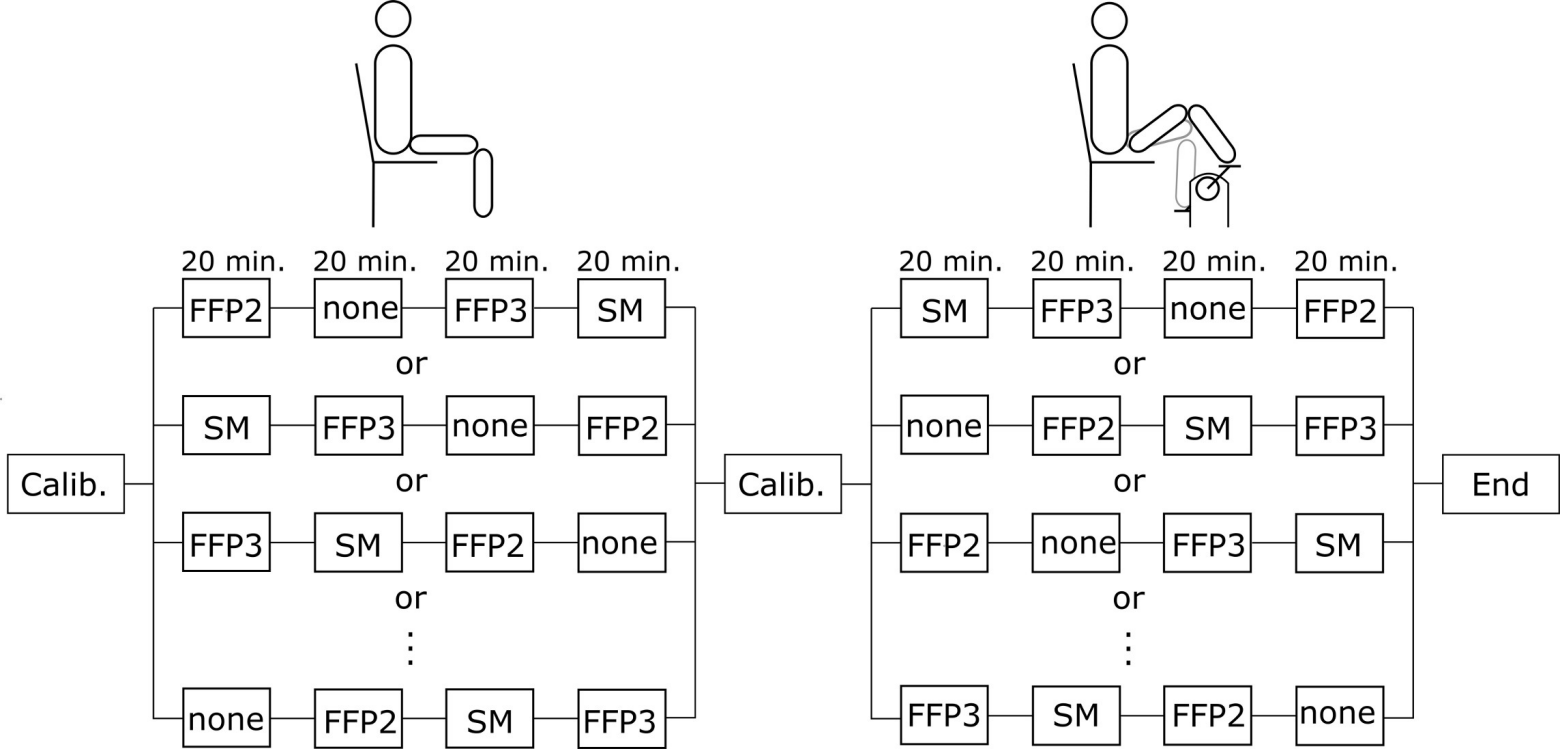
Schematic of measurement procedure. After a calibration measurement (Calib.) four facepiece conditions (no facepiece (none), surgical mask (SM), FFP2 respirator, FFP3 respirator) were presented for 20 minutes in randomized order at rest. Subsequently another calibration measurement was performed and the four facepiece conditions order were presented, again in randomized order while physical activity was performed. Between the measurements subjects were allowed to pause and continue the measurements at their own discretion.

All research was performed in accordance with all relevant national regulations and institutional policies and according to the Declaration of Helsinki. The study was approved by the Ethics Committee of the University of Freiburg (No. 20–1207) and registered before inclusion of the first subject at the German Register of Clinical Studies (DRKS00024135). All participants had given full written informed consent. Measurements were conducted between February 22, 2021 and May 01, 2021 in a laboratory room of 20 m^2^, air conditioned to 21°C and relative humidity of 40%. A total of 24 lung healthy adult subjects were involved in this study. They were asked to breathe without a facepiece, with a surgical mask (1A3100 Earloop 3-PLY, Dochem), a FFP2 respirator (TY0929V, Te Yin or atemious pro Art. 2001, Univent Medical) and a FFP3 respirator (silv-Air 7312, uvex) during rest and during physical activity (Fig 2). The latter was achieved through exercising on a recumbent ergometer at 50 to 100 W (ergometrics ER900 EL, Ergoline GmbH, Bitz, Germany). The choice of facepieces used for this part of the study was based on the availability of the respective facepiece type at that time. The subjects were instructed to independently place the facepieces on their faces, ensuring that their mouths and noses were fully covered, and to adjust the metal clip to contour their individual nose shapes. They were also encouraged to fasten the head straps themselves, if the facepieces provided such straps, in order to optimize the fit of the face masks. This method of having participants self-apply the facepieces should represent the way the general public commonly uses the facepieces. All experiments in a subject were performed in one session. To exclude effects of fatigue, the four measurements during rest were always performed first, and followed by the four measurements during physical activity. Between the measurements, subject were allowed to take pauses and to continue at their own discretion. For the measurements at rest and during physical activity, facepiece conditions, including the no facepiece condition, were randomly assigned according to a randomization sequence, previously computer-generated without measures for counterbalancing. Each measurement condition was maintained for 20 minutes.

Electrical impedance tomography (EIT) was utilised for measuring tidal volume and breathing frequency without the necessity of breathing via a flow measuring device. Therefore, an electrode belt for EIT (PulmoVista 500, Dräger medical, Lübeck, Germany) was placed circumferentially around the thorax at the level of 4th to 5th intercostal space. Volume calibration of the EIT signal was performed as described elsewhere [7]. In brief, before the study measurements, EIT data were recorded synchronously to flow measurements via a pneumotachograph (Fleisch Type 2, Dr. Fenyves & Gut, Hechingen, Germany) and the amplitude of the sum signal of the impedances was aligned to the correspondingly determined breathing volume.

SaO_2_, heartrate and oscillometric blood pressure at the left upper arm were recorded from an intensive care monitor (Sirecust 1281, Siemens Medical Electronics, Danvers, MA) and transcutaneous PCO_2_ (PCO_2_) was recorded from a digital monitor (V-Sign, SenTec AG, Therwil, Switzerland).

At the end of each measurement the subjects rated the discomfort of breathing on a continuous graphical discomfort scale ranging from 0 (easy) to 10 (intolerable).

In the subsequent offline analysis, minute volumes were determined from measured tidal volumes and breathing frequency and the facepiece-related additional minute volume was calculated as the difference between minute volume with facepiece and minute volume without facepiece, respectively. Further, the theoretical additional minute volume, required to compensate for rebreathed volume was calculated as the product of the respective respiratory rate and rebreathed volume with the respective facepiece for conditions with and without ambient airflow.

### Statistical analyses

Data are given as mean ± SD if not indicated otherwise. For the analyses of rebreathed volumes in the model study we used linear mixed model analysis with facepiece type, tidal volume and ambient airflow as factors.

For statistical analyses of data from subjects we used mixed model analysis with facepiece type and rest/exercising condition as fixed effect and subject as random coefficient, followed by post hoc test with Bonferroni correction. Under the assumption of normal distribution one can estimate from the normal range of PCO_2_ of 34 to 46 mmHg its SD as 3 mmHg. Regarding the matched pairs design for the post hoc comparison and an expected effect of 2.5 mmHg, a total sample size of 24 would be required to achieve a test power of 0.8 at an alpha level of 0.05, corrected for 12 comparisons (4 facepiece conditions at rest = 6 comparisons and 4 facepiece conditions when exercising = 6 comparisons).

## Results

### Bench study

The amount of rebreathed volume depended on the facepiece type (p < 0.001) and increased with the protection class (Table 2). Rebreathed volume was higher with higher tidal volume (p < 0.001).

**Table 2:** Volumes of effectively rebreathed air caused by surgical masks and respirators for simulation of breathing with low and high tidal volume (VT). Bold figures indicate measurements for which end-inspiratory CO_2_ partial pressure remained > 10% of end-expiratory PCO_2_.

		VT = 450 ml		VT = 950 ml	
Mask		Ambient airflow		Ambient airflow	
Type	Protection class	off	on	off	on
					
1A3100	Surgical mask	49 ± 1	-3 ± 1	101 ± 9	-4 ± 6
Aura 1862+	FFP2 respirator	**112 ± 1**	56 ± 1	229 ± 8	113 ± 1
Aura 1872V+	FFP2 (EV) respirator	**115 ± 2**	73 ± 1	209 ± 7	98 ± 5
TY0929V	FFP2 (EV) respirator	**68 ± 1**	24 ± 1	149 ± 11	21 ± 8
atemious pro	FFP2 respirator	45 ± 2	-1 ± 1	144 ± 3	22 ± 4
Aura 1863+	FFP3 respirator	**109 ± 1**	76 ± 2	233 ± 3	120 ± 9
silv-Air 7312	FFP3 (EV) respirator	**92 ± 12**	**76 ± 2**	247 ± 1	164 ± 1

FFP2/FFP3 = respirator according to respective protection class. (EV) = mask with expiratory valve. Data are given as mean ± SD. The end-inspiratory and end expiratory CO_2_ partial pressures of the respective measurements are given in S2 File.

Ambient airflow reduced the rebreathed volumes provided by surgical masks and respirators on average by 17% to 100% (all p < 0.001). This effect was smaller in facepieces with higher protection class. Particularly with the surgical mask and with two FFP2 respirators ambient airflow reduced rebreathed volume to less than 25 ml at both tidal volume conditions.

At low tidal volumes > 10% of rebreathed gas remained in the inspired air until the end of inspiration for most respirators but not for the surgical mask. For high tidal volumes this was found for no facepiece.

### Study in healthy subjects

One subject interrupted the measurement due to pain in the knee during pedalling. Data from this subject were not included in the data analyses. The included subjects’ (10 male, 13 female) average age was 27.6 years, ranging from 21 to 59 years. All subjects were able to wear all types of facepieces at rest and when exercising. When exercising the subjects generated an ergometer power of 80.4 ± 13.8 W. Within the breaths of the calibration data, tidal volumes determined via EIT deviated from those determined from respiratory data with a relative error of 2.5 ± 1.7%.

PCO_2_ was increased when wearing facepieces and when exercising (both p < 0.001). The effects of facepieces on PCO_2_ were more pronounced at rest than during exercising (Table 3). Heartrate, blood pressures, and breathing frequency were increased during exercising (all p < 0.001) compared to rest but were not influenced by wearing facepieces (all p > 0.1). SaO_2_ was not modified by wearing facepieces (p = 0.442) but slightly lower when exercising (97.5 ± 1.1%) compared to at rest (98.2 ± 1.2%, p < 0.001). Tidal volume and minute volume were higher when exercising (both p < 0.001) and increased with increasing protection class of the facepiece (both p < 0.001). The additional minute volume during breathing with facepieces was in the range of the theoretical additional minute volume caused by the respectively determined rebreathed volume during rest but higher during exercising (Table 3).

**Table 3:** Physiological variables during breathing without and with masks.

	Mask	HR [1/min]	RR [mmHg]		SaO_2_ [%]	PCO_2_ [mmHg]	VT [ml]	f [1/min]	MV [l/min]	addMV [l/min]	add MVth [l/min]
			sys	dia							
**Rest**											
	no mask	65 ± 8	118 ± 10	70 ± 8	98.4 ± 1.3	35.6 ± 4.2	682 ± 335	13 ± 5	8.0 ± 2.4	-	-
	SM	64 ± 8	118 ± 10	69 ± 8	98.1 ± 1.3	37.0 ± 4.6	681 ± 286	13 ± 4	8.1 ± 1.6	0.1 ± 2.1	0 to 0.7
	FFP2	66 ± 9	118 ± 10	68 ± 8	98.3 ± 1.2	37.5 ± 3.9*	709 ± 272	13 ± 3	8.4 ± 2.0	0.4 ± 2.0	0.3 to 0.8
	FFP3	65 ± 7	119 ± 11	71 ± 8	98.0 ± 1.2	38.8 ± 3.6*^#^	744 ± 295	13 ± 4	9.0 ± 1.6	1.0 ± 2.4	1.1 to 1.3
**Exercising**											
	no mask	132 ± 13	139 ± 18	66 ± 9	97.8 ± 0.9	38.1 ± 3.5	1011 ± 194	28 ± 6	27.3 ± 5.5	-	-
	SM	132 ± 14	141 ± 18	65 ± 12	97.7 ± 1.2	38.5 ± 3.1	1140 ± 216	28 ± 6	31.1 ± 6.5	3.8 ± 2.8	0 to 2.8
	FFP2	136 ± 15	144 ± 15	67 ± 10	97.6 ± 1.0	38.8 ± 3.1	1313 ± 362*	26 ± 7	32.6 ± 6.5	5.3 ± 4.2	0 to 3.8
	FFP3	135 ± 15	145 ± 18	66 ± 8	97.8 ± 1.2	39.5 ± 3.3	1325 ± 353*	27 ± 6	33.9 ± 6.3	6.6 ± 3.7	4.3 to 6.6

SM = surgical mask, FFP2/FFP3 = respirator according to respective protection class, HR = heartrate, RR = systolic (sys) and diastolic (dia) blood pressures according to Riva-Rocci, PCO_2_ = transcutaneous blood carbon-dioxide pressure, VT = tidal volume, f = breathing frequency, MV = minute volume, add MV = additional MV to without mask, add MVth = range of theoretical additional MV required to compensate dead-space of the mask = f × rebreathed volume (Table 1) without to with ambient airflow.

* = p < 0.05 compared to no mask

^#^ = p < 0.05 compared to SM at same rest/work condition in Bonferroni corrected post hoc test. Data are given as mean ± SD.

Discomfort of breathing increased with protection class of the facepieces and was perceived higher when exercising than at rest (Table 4). In no case did a subject rate discomfort of breathing as intolerable or interrupt the ongoing measurement due discomfort of breathing resulting from the presence of a facepiece.

**Table 4:** Discomfort of breathing at rest and during exercising on a recumbent ergometer rated on a graphical scale (0–10).

	Mask			
	none	SM	FFP2	FFP3
**Rest**	0.0 ± 0.2	1.2 ± 1.1*	2.3 ± 1.2*^#^	2.9 ± 1.6*^#^^§^
**Exercising**	0.6 ± 1.0	2.3 ± 1.2*	3.7 ± 1.4*^#^	4.6 ± 1.6*^#^

SM = surgical mask, FFP2/FFP3 = respirator according to respective protection class.

* = p < 0.05 compared to no mask

^#^ = p < 0.05 compared to SM

^§^ = p < 0.05 compared to FFP2 at same rest/work condition in Bonferroni corrected post hoc test. Data are given as mean ± SD.

## Discussion

The main findings of this study are that a) surgical masks and respirators showed a type-dependent amount of rebreathed air caused by the facepieces’ dead-space, b) the presence of ambient airflow significantly reduced the amount of rebreathed air caused by the facepieces’ dead-space, c) both during periods of rest and physical activity, lung-healthy subjects compensated for a facepieces’ deadspace by increasing tidal volume but not ventilation frequency.

The amount of rebreathed air increased generally with protection class, which we attribute to the higher number of filter material layers and more voluminous design of the higher protection class facepieces. The latter may be potentially required to reduce total airway resistance of the facepiece in respect to the denser material.

Ambient airflow reduced the amount of rebreathed air for all facepieces to an unexpected amount. We attribute this to two effects: First, ambient airflow maintains PCO_2_ close to 0 on the ambient surface of the respective facepiece preventing from rebreathing of CO_2_ that has not diffused from the facepieces’ surface. Second, ambient airflow partly penetrates the facepiece’s filter material and generates certain washout of expired air inside the facepiece’ lumen. The higher number of layers in facepieces of higher protection class and their more voluminous design can reduce this air penetration to some extent. Consequently, fewer and less dense filter layers allow more washout of expired air in case of ambient airflow. This effect may be amplified by “leakage”-flow passing the facepieces’ filter material. Particularly with surgical masks noticeable amounts of air bypass the filter material during breathing [5,8]. Therefore, we assume that with the surgical mask and the flat-fold FFP2 respirators, ambient airflow caused a nearly total washout of expired air resulting in the elimination of nearly all effects of the facepieces’ dead-space.

**Fig 3:**
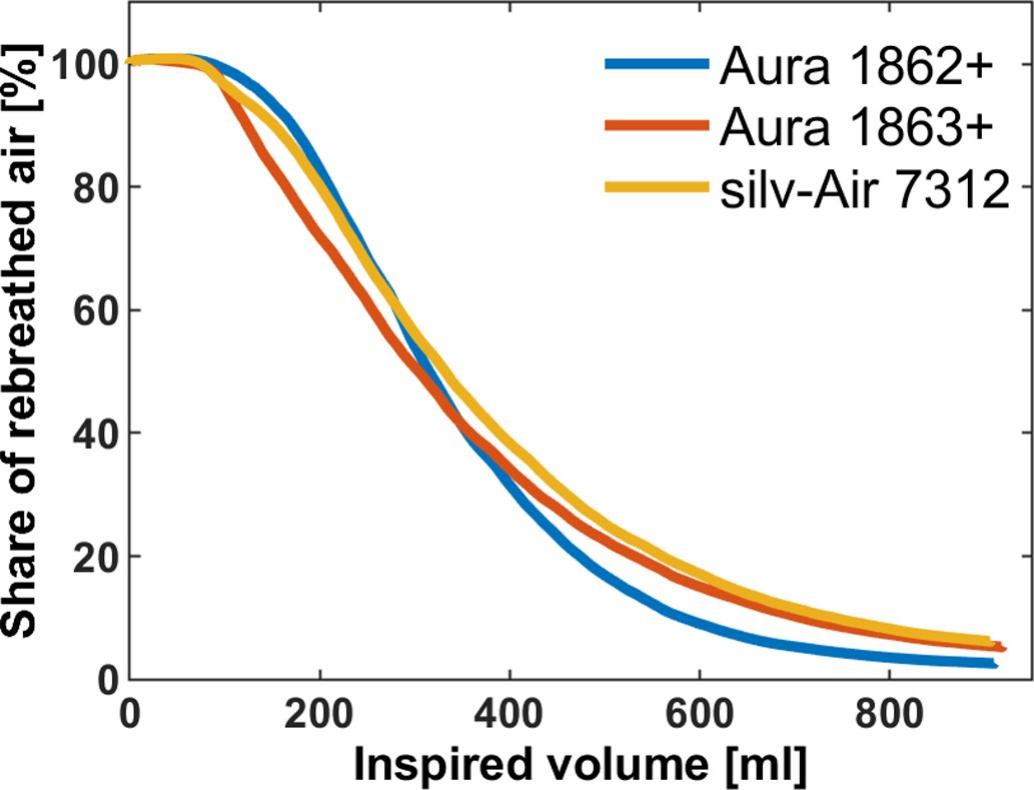
Exemplary curves for rebreathed air against inspired volume. The three facepieces show different characteristics in early and late shared of rebreathed air. While the Aura 1863+ (FFP3, 3M) shows a fast decline in the early phase of inspiration the decline is slower in the later phase. The Aura 1862+ (FFP2, 3M) shows an early delayed decline and a faster decline in the middle and late phases of inspiration. The silv-Air 7312 (FFP3, uvex) shows a slow decline in both, the early and the late phase of inspiration.

The anatomical dead-space in a healthy adult, attributed to the biological airways, is 2 ml/kg, i.e. 150 ml, thus it is about a third of a normal tidal volume [9]. Without ambient airflow the surgical mask increased volume of rebreathed air by up to 67% and FFP2 and FFP3 respirators increased volume of rebreathed air by up to 137% and 163%, respectively. Thereby, dead-space and volume of rebreathed air need to be distinguished. Noticeable amounts of rebreathed air persisted in the inspiration gas while the total dead-space within the system (facepiece + tubing without facepiece) was much smaller than inspiration volume. Initially this may be surprising. However, it is important to consider that during inspiration, ambient air enters the space inside the facepiece and mingles with the gas from the preceding expiration. As a result, mixed gas with a progressively decreasing portion of rebreathed air is inspired. The process of gas mixing and subsequent reduction of rebreathed gas during inspiration exhibited variations across different facepieces (Fig 3). These variations appear to be dependent on two key factors: protection class and the facepiece’s design type, i.e. the flat-fold (atemious pro Art. 2001 and all Aura) or the cup-shaped type (TY0929V and silv-Air 7312). Further, it is important to consider the total dead space of the system in this context, which encompasses the dead space of the tubing system to our measuring point which was designed to closely mimic that of an adult person.

In line with earlier studies [10], blood carbon pressure increased with increasing protection class during breathing via facepieces. Interestingly, this was less pronounced during physical activity. The subjects exhibited a notable ability to effectively compensate for the presence of additional dead space. However, it appears that at rest they rather tolerated the small increase of PCO_2_ instead of further compensating it by increasing minute volume. It is to note that when considering the physiological range of arterial CO_2_ partial pressure as 35 to 45 mmHg, our subjects remained well below the upper limit. In summary, and again in line with earlier studies [11–14] we have reason to assume that the observed facepiece-dependent increase in PCO_2_ can be considered physiologically irrelevant.

While at rest, subjects compensated quite well the additional minute volume required to adapt to the rebreathed volume caused by the facepiece. However, they rather overcompensated the theoretically required minute volume during physical activity, particularly when considering ambient airflow. We can only speculate about this observation: On one hand, the calculation of theoretically required minute volume is a comparably rough estimate and the found deviations from of measured additional minute volume and the range of the theoretically required additional minute volume (< 5% of measured minute volume) may partly be attributed to measurement precision. On the other hand, it has to be considered that compensating rebreathed volume required an increased effort of breathing, and facepieces additionally increase airway resistance depending on protection class. Consequently, more energy is required to sustain a higher breathing frequency and overcome the additional airway resistance. This results in an increased work of breathing, subsequently raising metabolism, and requiring additional compensation of CO_2_ elimination.

It is to note that other studies found adverse effects of wearing facepieces in terms of increased PCO_2_ and decreased PO_2_ [15], altered metabolism [16] and particularly in children discomfort and headaches were reported [17]. According to our study, also in adults [18], discomfort of breathing increased with the protection class of the respective facepiece and was in our study notably more pronounced during physical activity than at rest. However, breathing discomfort remained well within tolerable range. In summary, our findings concerning the ability to compensate for the facepieces dead-space do not relate to the public debate on the discomfort caused by wearing facepieces. This observation is particularly relevant given the fact that FFP3 respirators were never required in public areas.

### Limitations of the study

Generally, bench studies cannot fully reproduce all details of the real world. We simulated expiratory air by mixing CO_2_ into ambient air. Human expiration gas has a temperature of 37°C and is moisturised to nearly 100% which may change the facepieces’ resistance [19]. We chose not to simulate these conditions in our bench study as we believed that this would not enhance the accuracy of our determination of rebreathed air volume. Further, the simulated CO_2_ partial pressures of mixed gas were not always in the physiological range. Achieving precise gas mixing in a non-static system requires meticulous fine-tuning to maintain a sufficient flow balance. Therefore, we accepted non-physiological gas mixtures within the range that could be accurately measured by our sensor. Considering the principle of determination of rebreathed volume via Bohr’s approach, this should not have impacted our measurements. The face mannequin did not include modelling of a soft skin texture. This may have influenced the washout effects of ambient airflow. Finally, we did not take measures for optimally fitting the facepieces to the contour of the face mannequin. Consequently, air tightness between facepiece and face mannequin was not optimized. However, the variances of our measurements were low, indicating comparable tightness conditions and thus reproducibility among the measurement repetitions. Furthermore, we feel that by omitting special measures to improve sealing, we have reproduced more realistic conditions of using a facepiece in a non-clinical setting than if we had artificially enhanced air tightness.

For obvious reasons, the study in subjects could not be performed in a blinded design. The knowledge about wearing a facepiece may have modified the breathing behaviour of the subjects beyond the effects resulting from the facepieces’ dead-space. However, in doing so, we aimed to mirror the realistic condition in which a person wearing a facepiece is indeed aware of this fact.

Two different FFP2 respirators were used in the study in subjects. Due to the unexpected shortage of protection equipment caused by the COVID-19 pandemic, we had to switch the type of facepiece to the available supply. One of both included an exhalation valve, which is considered to potentially impact the build-up of CO_2_, heat and humidity within the facepiece, as all exhaled air should be directed through the valve rather than through the filter. However, we had determined comparable properties for both respirator types (Table 2), thus we feel justified to argue that this had not influenced our findings to a relevant extent.

For ethical reasons, application of an arterial line for the purpose of our study would not be justifiable. Transcutaneous PCO_2_ measurement gives a reliable estimate of arterial PCO_2_ measured via blood gas analysis but responds with delay on rapid changes [20]. We considered the response time by keeping every measurement condition for 20 minutes.

In our study we solely focused on the impact of wearing facepieces. Also environmental conditions and physiology of breathing interact with each other. It was shown that exposure to CO_2_ and other bioeffluents modulated vital parameters and mental performance [21] and restricted breathing physiology [22]. Moreover, metabolism and thus CO_2_ production itself was modulated by time of day [23] and higher temperature was associated with increased CO_2_ production [24]. Further studies are required to investigate if this can be exploited to further improve facepiece breathing, if required.

## Conclusion

Surgical masks and FFP2 and FFP3 respirators led to an increase in the amount of rebreathed air dependent on protection class and design. Lung-healthy adult subjects experienced the presence of all facepieces as well tolerable both during rest and physical activity, and compensated for the rebreathed air by increasing tidal volume rather than altering breathing frequency. The presence of ambient airflow may significantly reduce the burden when breathing through facepieces. This suggests that if facepieces are required during quiet sitting, e.g. in an office work environment, the presence of ambient airflow may decrease requirement of facepiece related dead space compensation.

## Supporting information

S1 File Images of the facepieces used in the study. From top to bottom: Surgical mask Dochem, FFP2 respirator 3M, FFP2 respirator Te Yin, FFP2 respirator atemious, FFP3 respirator uvex. Left: View from the front, right: View from the rear. Due to geometrical similarities only one type of facepiece is shown for each manufacturer.
(PDF)

S2 File Table of end-inspiratory and end-expiratory partial pressures of bench study. (PDF)

S3 File Raw data of the bench study. (XLSX)

S4 File Raw data of study in subjects. (XLSX)
