# Supplementary material for: Effective volume of rebreathed air during breathing with facepieces increases with protection class and decreases with ambient airflow
Source: PLoS One. 2024 Mar 21;19(3):e0299919. doi: 10.1371/journal.pone.0299919 (PMC10956828; doi:10.1371/journal.pone.0299919)
Supplement: S1 File — From top to bottom: Surgical mask Dochem, FFP2 respirator 3M, FFP2 respirator Te Yin, FFP2 respirator atemious, FFP3 respirator uvex. Left: View from the front, right: View from the rear. Due to geometrical similarities only one type of facepiece is shown for each manufacturer. (PDF) [file pone.0299919.s001.pdf]

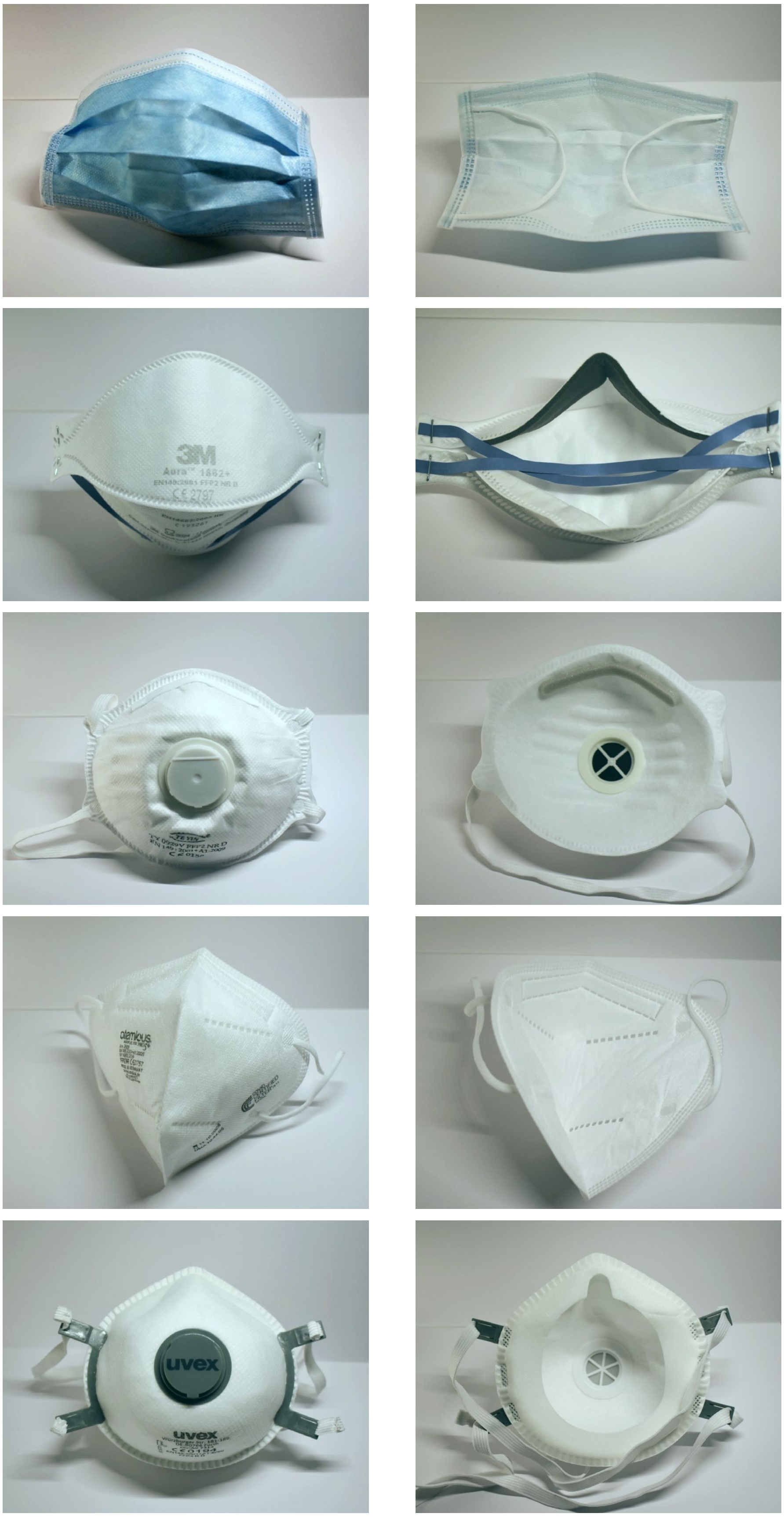

Supporting File1: Images of the facepieces used in the study. From top to bottom: Surgical mask Dochem, FFP2 respirator 3M, FFP2 respirator Te Yin, FFP2 respirator atemious, FFP3 respirator uvex. Left: view from the front, right: view from the rear. Due to geometrical similarities only one type of facepiece is shown for each manufacturer.
