# Supplementary material for: Effective volume of rebreathed air during breathing with facepieces increases with protection class and decreases with ambient airflow
Source: PLoS One. 2024 Mar 21;19(3):e0299919. doi: 10.1371/journal.pone.0299919 (PMC10956828; doi:10.1371/journal.pone.0299919)
Supplement: S2 File — (PDF) [file pone.0299919.s002.pdf]

Supporting File 2. CO_2 partial pressures at end of inspiration ($PieCO_2$) and end of expiration ($PieCO_2$) caused by surgical masks and respirators for simulation of breathing with low and high tidal volume (VT).

**Mask Type**	**Protection class**	**VT = 450 ml**				**VT = 950 ml**			
		**Ambient airflow**				**Ambient airflow**			
		**off**		**on**		**off**		**on**	
		**$PieCO_2$**	**$PeeCO_2$**	**$PeiCO_2$**	**$PeeCO_2$**	**$PieCO_2$**	**$PeeCO_2$**	**$PeiCO_2$**	**$PeeCO_2$**
1A3100	Surgical mask	4.3 ± 0.4	58.6 ± 1.7	0.4 ± 0.0	46.1 ± 0.7	2.1 ± 0.2	53.5 ± 1.2	0.3 ± 0 0	45.0 ± 0.5
Aura 1862+	FFP2 respirator	**18.8 ± 0.5**	88.6±2.0	5.2 ± 0.2	66.6 ± 1.0	1.8 ± 0.1	70.4 ± 0.7	0.4 ± 0.0	59.82 ± 1.3
Aura 1872V+	FFP2 (EV) respirator	**16.1 ± 0.5**	90.3± 0.5	5.6 ± 0.3	70.6 ± 0.4	2.7 ± 0.0	68.2 ± 1.4	0.4 ± 0.1	53.6 ± 0.5
TY0929V	FFP2 (EV) respirator	**7.1 ± 0.0**	64.1 ±0.5	1.8 ± 0.0	50.1 ± 0.5	3.1 ± 0.4	58.4 ± 1.4	0.3 ± 0.1	46.4 ± 0.5
atemious pro	FFP2 respirator	5.4 ± 0.2	60.9 ± 0.4	0.9 ± 0.1	50.3 ± 0.3	2.2 ± 0.2	51.6 ± 0.4	0.3 ± 0.0	41.3 ± 0.2
Aura 1863+	FFP3 respirator	**16.7 ± 0.1**	85.4 ± 1.2	6.7 ± 0.3	70.3 ± 0.5	3.4 ± 0.2	64.2 ± 0.8	0.7 ± 0.1	50.9 ± 2.1
silv-Air 7312	FFP3 (EV) respirator	**16.3 ±2.1**	72.2 ± 3.0	**9.4 ±0.4**	68.3 ± 0.9	4.5 ± 0.7	74.1 ± 1.4	1.5 ± 0.0	62.2 ± 0.2

FFP2/FFP3 = respirator according to respective protection class. (EV) = mask with expiratory valve. Data are given as mean ± SD.
